# Perceived employability as a dual resource: modeling career success during the university-to-work transition

**DOI:** 10.3389/fpsyg.2026.1747952

**Published:** 2026-05-01

**Authors:** Ringo Moon-ho Ho, Kim-Yin Chan, Marilyn A. Uy, Jeffrey C. Kennedy

**Affiliations:** 1 Psychology Programme, School of Social Sciences, Nanyang Technological University, Singapore, Singapore; 2 Nanyang Business School, Nanyang Technological University, Singapore, Singapore; 3 Massey Business School, Massey University, Auckland, New Zealand

**Keywords:** career competencies, career distress, conservation of resources theory, perceived employability, person–job fit, subjective career success, university-to-work transition, proactive personality

## Abstract

**Introduction:**

In dynamic labor markets, universities are increasingly expected to foster not only immediate employment outcomes but also sustainable career development among graduates. Drawing on conservation of resources theory, this study examines how perceived employability at graduation influences subjective career success during the university-to-work transition via two pathways: employment quality (person–job fit) and psychological wellbeing (career distress).

**Methods:**

Using a three-wave longitudinal design, data were collected from 385 Singaporean graduates at graduation, 1 year post-graduation, and 2 years post-graduation. The hypothesized relationships were tested using cross-lagged structural equation modeling.

**Results:**

Perceived employability at graduation was positively associated with person–job fit and negatively associated with career distress 1 year later, and these variables fully mediated the effect of perceived employability on subjective career success 2 years post-graduation. Proactive personality and career competencies were confirmed as antecedents of perceived employability and career distress, highlighting the role of both trait and learned resources in employability development.

**Discussion:**

These findings suggest that enhancing graduates’ perceived employability can promote better employment fit, reduce career-related distress, and ultimately foster higher subjective career success, with implications for career education, policy, and organizational practices in recruitment, onboarding, and early job design. In addition, we introduce a diversified investment metaphor that conceptualizes perceived employability as a psychological portfolio balancing short-term employment fit and long-term career resilience, offering a heuristic for future research on strategic resource allocation during the university-to-work transition.

## Introduction

The transition from university to the workforce—commonly referred to as the university-to-work transition—is a critical developmental phase for graduates, marked by uncertainty, identity formation, and career decision-making (Ng and Feldman, 2007; Akkermans et al., 2021). As global labor markets become increasingly dynamic and protean, universities are expected not only to facilitate employment outcomes but also to foster sustainable career development (Jackson and Bridgstock, 2018; Tomlinson, 2012). This shift has prompted growing interest in psychological indicators of career readiness and success, particularly perceived employability (PE), person–job fit (P–J fit), career distress, and subjective career success (SCS).

Among these, PE, which is defined as an individual’s belief in their ability to obtain and maintain employment (Berntson and Marklund, 2007), has emerged as a critical psychological resource during the university-to-work transition. Unlike objective employability, which focuses on skills or qualifications, PE captures individuals’ self-appraisal of their labor market value and future prospects (Fugate et al., 2004; Vanhercke et al., 2014). Recent longitudinal research shows that PE evolves during the university-to-work transition, reinforcing its conceptualization as a dynamic resource shaped by both personal and contextual factors (Grosemans et al., 2023). PE has been shown to buffer against job insecurity and psychological strain (Harari et al., 2023) and to influence job search behavior, employment quality, and wellbeing (Berntson et al., 2008; De Cuyper et al., 2012a,b).

Despite its relevance, the PE–SCS relationship remains underexplored, particularly in longitudinal university-to-work transition contexts. While SCS, which is defined as individuals’ self-evaluation of their career achievements (Greenhaus et al., 1990; Shockley et al., 2016) is increasingly recognized as a key outcome of career development, studies often treat PE and SCS as isolated constructs or examine them cross-sectionally (Lo Presti and De Rosa, 2024; Seibert et al., 2024). Moreover, existing research tends to conflate employability with employment, overlooking the nuanced ways in which PE may shape both immediate job outcomes and longer-term career satisfaction (Fugate et al., 2021; Lo Presti and Pluviano, 2016).

This study addresses these gaps by modeling PE as a dual psychological resource that influences SCS through two distinct pathways: employment quality (via person–job fit) and psychological wellbeing (via reduced career distress). Person–job fit, defined as the alignment between individual capabilities and job demands (Edwards, 1991; Cable and DeRue, 2002), reflects the quality of employment and has been linked to job satisfaction, engagement, and career advancement (Ng and Feldman, 2014). Career distress, on the other hand, captures emotional strain related to career decision-making and goal setting (Creed et al., 2016), and is particularly salient during the university-to-work transition when individuals face high uncertainty and limited experience.

These four constructs: PE, P–J fit, career distress, and SCS, are not only theoretically significant but also practically relevant for multiple stakeholders. For universities, they offer metrics for evaluating the effectiveness of career education beyond job placement. For students and graduates, they provide insight into how psychological resources shape early career outcomes. For employers, they highlight the importance of supporting employability and wellbeing to enhance retention and performance. For career counselors and policymakers, they offer a framework for designing interventions that promote both employment and career sustainability.

By integrating job/employment and career literatures and applying the conservation of resources theory (Hobfoll, 1989, 2001), this study offers a refined framework for understanding employability development during the university-to-work transition. Specifically, we conceptualize PE as a resource that can be invested across both job-specific and career-wide domains, and we propose a longitudinal model in which PE influences SCS via person–job fit and career distress. To complement this framework, we include two antecedents to PE and distress that capture distinct forms of resources particularly relevant at graduation. Proactive personality reflects a dispositional tendency to take initiative and influence one’s environment. Evidence suggests that broad personality traits tend to show substantial rank-order stability during young adulthood (Bleidorn et al., 2022), making proactive personality a meaningful baseline input in our model. Career competencies, in contrast, are more closely tied to learning, reflection, and career self-management. Longitudinal findings indicate that these competencies can strengthen through early work experiences and intentional developmental efforts (Grosemans and De Cuyper, 2021). Considering both constructs allows us to represent relatively enduring tendencies alongside capacities that may evolve during the university-to-work transition.

Recent longitudinal research further suggests that perceived employability and related career resources do not remain static during the move from graduation into full-time work (Grosemans et al., 2023). We therefore approach our model with this developmental possibility in mind and revisit the implications of such change in the Discussion.

### Theoretical framework: conservation of resources theory

To explain how these constructs interact during the university-to-work transition, we draw on the conservation of resources theory (Hobfoll, 1989, 2001), which posits that individuals strive to acquire, protect, and invest resources that support goal attainment and buffer against stress. Conservation of resources theory provides a robust framework for understanding how individuals manage psychological resources during career transitions, and has been widely applied to explain stress and adaptation in organizational contexts, including employability (De Cuyper et al., 2012a,b; Harari et al., 2023), burnout (De Cuyper et al., 2012a,b), and job insecurity (Fugate et al., 2021). Recent refinements to conservation of resources theory emphasize the dynamic interplay of resource gain and loss cycles, and the contextual factors that shape resource investment decisions (Halbesleben et al., 2014). However, its application to the university-to-work transition context remains limited.

While career self-management theory (Lent and Brown, 2013) emphasizes agentic processes such as goal setting, self-efficacy, and self-regulation in career development, conservation of resources theory (Hobfoll, 1989, 2001) offers a complementary perspective focused on resource dynamics—acquisition, protection, and investment. Career self-management theory is particularly useful for understanding how individuals proactively shape their career paths. In contrast, conservation of resources theory provides a broader framework for modeling how psychological resources like PE buffer against stress and support both immediate and long-term career outcomes. This makes conservation of resources especially suitable for examining how PE functions as a dual resource during the university-to-work transition, influencing both employment quality and psychological wellbeing.

The university-to-work transition is a critical phase where individuals face competing demands—securing employment while building long-term career resilience. Conservation of resources theory’s emphasis on resource acquisition, protection, and investment is particularly relevant for modeling how PE functions as a psychological asset during this transition. In our framework, PE functions as a psychological resource that can enhance both immediate employment quality (via P-J fit) and broader career wellbeing (via reduced career distress). Conservation of resources theory supports the inclusion of both person–job fit and career distress as mediators in the PE–SCS relationship. Career distress reflects a perceived lack of resources, such as clarity, confidence, and control over career decisions (Creed et al., 2016). Individuals with high PE are likely to experience lower distress because they perceive greater control and options in the labor market (Berntson and Marklund, 2007; Harari et al., 2023). Person–job fit, defined as the alignment between individual capabilities and job demands (Edwards, 1991; Cable and DeRue, 2002), reflects employment quality and is influenced by resource availability. Individuals with high PE are more likely to secure jobs that match their skills and preferences, leading to better fit and ultimately higher career satisfaction. By modeling both mediators, we clarify how PE operates across traditionally siloed domains and demonstrate its dual-resource nature. Such a dual-pathway logic also aligns with the Job Demands-Resources (JD-R) model, which posits that job resources (e.g., fit) buffer against strain and enhance wellbeing (Demerouti et al., 2001).

We thus extend conservation of resources theory by conceptualizing PE as a dual resource—supporting both job-specific and career-wide outcomes. We also suggest that this allows us to think of PE as a diversified psychological investment, akin to a financial portfolio that balances short-term liquidity with long-term growth (e.g., Markowitz, 1959). In this metaphor, graduates with high PE are not merely investing in securing a job but are also allocating psychological resources toward career resilience and satisfaction. For example, a graduate may choose a role that offers developmental opportunities and aligns with long-term goals, rather than accepting the first available position. While not empirically tested here, we later discuss how this metaphor offers a conceptual lens for understanding and studying how individuals strategically allocate resources to manage risk and optimize outcomes across both job and career domains.

### Research model and hypotheses

**Figure 1:**
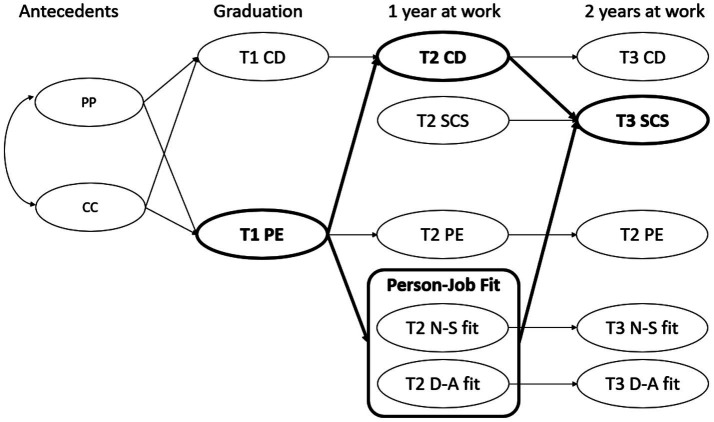
Hypothesized model of subjective career education outcomes during university-to-work transition with trait and competency antecedents and PE as a dual resource (or “diversified investment”) for SCS sub-model in bold. PP, Proactive Personality; CC, Career Competencies; PE, Perceived Employability; CD, Career Distress; NS-fit, Need-Supplies fit; DA, Demand-Abilities fit; SCS, Subjective Career Success.

We propose a prospective model (Figure 1) in which perceived employability (PE) at pre-graduation (T1) predicts subjective career success (SCS) 2 years later (T3) indirectly via T2 mediators: person–job (P–J) fit and career distress. The model yields four hypotheses (H1—H4):

*H1:* Higher T1 PE will prospectively predict lower T2 career distress, which will, in turn, predict higher T3 SCS (indirect effect via distress).

*H2:* Higher T1 PE will prospectively predict higher T2 P–J fit (needs–supplies and demands–abilities).

*H3:* T2 P–J fit will mediate the association between T1 PE and T3 SCS.

*H4:* T2 Career distress will mediate the association between T1 PE and T3 SCS.

In addition to modeling PE and distress as mediators, we include proactive personality and career competencies as antecedents to reflect input-based employability. Proactive personality represents a stable trait disposition to take initiative (Bateman and Crant, 1993; Seibert et al., 1999), while career competencies encompass learned skills and behaviors that support career development (Akkermans et al., 2013; Hirschi, 2012). This distinction aligns with the career resources model (Hirschi, 2012), which conceptualizes employability development as a function of both trait and learned resources. Prior research shows that career competencies enhance employability and career success through increased self-awareness and proactive career behaviors (De Vos et al., 2011), and that academic satisfaction and employability activities mediate their effects during the school-to-work transition (Lo Presti et al., 2022). Recent psychometric evidence also supports the distinction between resource-based and PE, reinforcing the view that PE functions as an output-based psychological resource shaped by underlying input-based factors (Lo Presti and De Rosa, 2024). By including these antecedents, our model offers a more comprehensive framework for understanding how individual differences and developmental resources shape employability and career outcomes.

## Materials and methods

### Participants and procedures

Our sample consisted of full-time, final year undergraduates from one of Singapore’s two large, comprehensive, research-intensive public universities. These universities offer undergraduate and postgraduate courses in various disciplines, ranging from engineering to arts, business, humanities, medicine, sciences, and social sciences. They are internationally competitive, having been ranked among the top 50 globally in various rankings in recent years.

Responding to recruitment emails sent out a month before graduation, 627 final year undergraduate students signed up to participate in the study, of whom 545 completed the Time 1 (T1) “graduation” survey with 484 passing the attention-check criterion. These respondents were invited to participate in a follow-up survey 1 year later; 433 completed the Time 2 (T2) survey with 421 (97.2%) passing our attention checks, and 399 completed the Time 3 (T3) survey with 100% passing our attention checks. Participants received SGD20 for completing the T1 survey, SGD25 for completing the Time 2 survey and SGD25 for completing the Time 3 survey. All questionnaires were administered in English. English is the primary language of higher education, government, and professional communication in Singapore.

P-J fit and SCS were measured after graduation (at T2 and T3), as these scales asked participants about their jobs and careers. As our research focused on undergraduates transitioning from university to work, we removed from the analysis 14 respondents who were not in full- or part-time employment at T2 or T3. The final samples comprised 385 recent graduates (240 females, 145 males, mean age 24.16, *SD* = 1.35) who were surveyed at the point of graduation and followed up 1 year later (T2), and then 2 years later (T3) in the workforce, with 93.5% working full time at T3. About 45.2% of these graduates majored in various STEM fields (engineering, mathematics and sciences), while the rest (54.8%) were from the fields of arts, humanities, business studies and social sciences. Approval for this research was obtained through the Institutional Review Board at [Nanyang Technological University] (Protocol numbers: 2022-246 and 2021-03-059).

### Analytic strategy

We used Mplus version 8.10 (Muthén and Muthén, 1998–2012) to conduct structural equation modeling with Maximum Likelihood (ML) estimation to test our hypotheses.

First, through confirmatory factor analysis (CFA), we tested measurement models by comparing: (a) for T1: a one-factor model (all items loaded on one factor) against a four-factor model (PE, career distress, career competencies and proactive personality as separate factors); (b) for both T2 and T3, respectively: a one-factor model against a five-factor model (PE, career distress, demand-abilities fit, needs-supplies fit and SCS). These analyses verified that the constructs investigated are distinct from each other.

Second, we assessed whether each scale functioned equivalently across waves before estimating structural paths. For each focal construct, we tested configural invariance (same pattern of factor–indicator relations across time) followed by metric invariance (equal factor loadings across time; Meredith, 1993). Because our research questions do not involve comparing latent means across time, metric invariance was sufficient for our purposes, and scalar invariance (equal item intercepts) was not required and therefore not pursued. Invariance was tested for each measure given its measurement schedule (two waves for needs–supplies and demands–abilities fit and for subjective career success; three waves for perceived employability and career distress).

Third, to test for the hypothesized mediation, we fitted a structural equation model with first-order autoregressive paths for each focal variable,^1^ e.g., PE at T1 predicting PE at T2 which in turn predicted PE at T3. We modeled cross-lagged paths among focal variables between successive time points, from earlier time point to later time point, i.e., PE at T1 predicting career distress, demands-abilities fit, needs-supplies fit and SCS at T2, PE at T2 predicting career distress, demands-abilities fit, needs-supplies fit and SCS at T3, demands-abilities fit at T2 predicting PE, career distress, needs-supplies fit and SCS at T3, etc. To test mediation (Hypotheses 3 and 4), we used bias-corrected bootstrapping with 1,000 resamples to obtain confidence intervals for indirect effects.

Model fit was assessed by the chi-square statistic, comparative fit index (CFI), root-mean-square error of approximation (RMSEA) and standardized root mean squared residual (SRMR). CFI and RMSEA are relatively unaffected by estimation technique with multivariate normal data (Cangur and Ercan, 2015), while SRMR is highly sensitive to mis-specified structural model components (Fan and Sivo, 2005). CFI measures the incremental fit between the hypothesized model and a null model where all observed variables are uncorrelated, while RMSEA is an adjusted measure of absolute fit based on the complexity of the hypothesized model. In this study, we adopted Hu and Bentler’s (1999) two-index presentation strategy, where SRMR ≤ 0.08 and (CFI ≥ 0.95 OR RMSEA ≤ 0.06) indicate acceptable model fit.

### Common method variance

Common method variance (CMV) refers to variance attributable to the measurement method rather than to the constructs the measures represent and is a concern when self-report surveys are used to collect data simultaneously from the same group of participants. Various design and statistical approaches have been proposed in checking and reducing the threat of CMV (see Podsakoff et al., 2003 for example). One way is to have temporal separation (see Menard, 2008) between the predictor and criterion variables. Temporal separation is viewed favorably in behavioral research, and there is strong evidence that it can reduce the threats of CMV (e.g., Lindell and Brandt, 2000). Since our study adopted a longitudinal design with the antecedents, mediators and outcome variables temporally separated, CMV does not pose a major threat to our findings.

### Focal measures: subjective work-related outcomes of university education

*Perceived employability (PE):* This was measured at all three time points using six items, five items from Berntson and Marklund (2007) and one item from Berntson et al. (2008). The scale measures a respondent’s PE based on their perceived skills, experience, network, personal traits, and knowledge of the labor market. Participants were asked to rate their level of agreement using a five-point scale on how well each statement described them. Examples were: “My competence is sought-after in the labor market”, and “I know of organizations/companies where I can get work”. A single factor model was fitted to the PE responses at each time point separately and with comparable goodness of fit over the three time points (T1/T2/T3): CFI = 0.921/0.955/0.902; RMSEA = 0.061/0.057/0.064; and SRMR = 0.043/0.035/0.050. Scale scores were formed by averaging the 6 items at each time point. Cronbach’s alphas were 0.804, 0.819 and 0.802 at T1, T2 and T3, respectively.

*Career distress*: This was measured at all three time points using nine items from Creed et al. (2016) assessing negative feelings toward the process of career decision-making (e.g., stress, helplessness, anxiety). Past analyses revealed a unidimensional factor underlying the scale, with high reliability. In the current study, participants were asked to rate their level of agreement using a five-point scale on how well each statement described them. A sample item: “I feel stress or pressure to select a satisfying career.” For each time point, a single factor model fitted well with comparable goodness of fit over the three time points (T1/T2/T3: CFI = 0.994/0.979/0.962; RMSEA = 0.026/0.053/0.067); and SRMR = 0.023/0.029/0.034. Cronbach’s alphas were 0.815, 0.849 and 0.821 at T1, T2 and T3, respectively.

*Person-Job (P-J) fit*: The self-reported P-J fit scale originally consisted of three-item distinct subscales to assess perceived person-organization fit, needs-supplies fit, and demands-abilities fit (Cable and DeRue, 2002), with a high level of convergent and discriminant validity, and reliability. For the current study, person-organization fit was not the focus and was thus excluded. P-J fit comprises two correlated factors: Needs-supplies fit which concerns how well the job satisfies the individual’s needs; and demand-abilities fit which concerns how well the individual’s skills are in demand or relevant to the job (see Kristof-Brown et al., 2005). Thus, in this research, P-J fit represents the interplay between job and person factors in the worker’s current employment, a topic which has been somewhat neglected in more agentic, careers research on PE. At T2 and T3, we only used six items, with three items each for demand-abilities fit (e.g., “My abilities and training are a good fit with the requirements of my job”) and needs-supplies fit (e.g., “The job that I currently hold gives me just about everything that I want from a job”). Participants were asked to rate their level of agreement using a seven-point scale on how well each statement described them. At both T2 and T3, a correlated two-factor model fitted the data well (one factor for the demands-abilities fit and one for the needs-supplies fit): CFI = 0.982 and 0.992, RMSEA = 0.076 and 0.062, SRMR = 0.031 and 0.020. Scale scores were computed by averaging the three items within each person-fit subscale. Cronbach’s alphas at T2 and T3 were (i) 0.832 and 0.882 for the three-item needs-supplies fit scale, and (ii) 0.805 and 0.858 for the three-item demand-abilities fit scale.

*Subjective Career Success (SCS):* Considering recent calls for more multidimensional measures of SCS, Seibert et al. (2024) advised that it is still acceptable to use short, unidimensional or global measures of SCS in research studying the general notion of SCS. We therefore used five items from Greenhaus et al. (1990) to measure SCS measured at T2 and T3. The five-item scale was shown to have good quality of construct validity and reliability (e.g., see Seibert et al., 1999). Participants were asked to rate their level of agreement using a 5-point scale on how well each statement described their satisfaction with career success. A sample item was: “I am satisfied with the success I have achieved in my career.” CFA showed that a one-factor model fitted the current data at T2 and T3 well (CFI = 0.968 and 0.997; RMSEA = 0.077 and 0.040; SRMR = 0.033 and 0.013). Scale scores were computed by averaging the five items with Cronbach’s alpha of 0.839 and 0.883 at T2 and T3, respectively.

### Measures of antecedents

*Career competencies*: Akkermans et al.’s (2013) 21-item Career Competencies Questionnaire (CCQ) was administered to the participants at T1, i.e., point of graduation from university. The CCQ measured 6 dimensions, best represented by a second order factor model with 6 dimensions as first order factors. Akkermans et al. validated the scale with samples of young employees (aged 16–30), comparable with our sample. Participants were asked to indicate on a five-point scale the degree to which they agreed with statements about the development of their careers, i.e., their work in the present and future. Sample items (by dimensions) included: “I know what I like in my work” (reflection on motivation); “I know my strengths in my work” (reflection on qualities); “I can clearly show others what my strengths are in my work” (self-profiling); “I know how to ask for advice from people in my network” (networking); “I am able to explore my possibilities on the labor market” (work exploration); “I can make clear career plans” (career control). In our study, CFA showed a good fit for a model with a higher order factor underlying the six subscales (CFI = 0.927, RMSEA = 0.056, SRMR = 0.050). Subscale scores were computed according to each of these domains. A global scale score was then computed by averaging the six subscale scores with Cronbach alpha of 0.912.

*Proactive personality*: To manage survey length at T1, a 10-item version of Bateman and Crant’s (1993) Proactive Personality scale developed by Seibert et al. (1999) was administered to the participants at T2 rather than T1, on the basis that it is a relatively stable trait. The short version was validated with both university and working samples in the literature with reliability comparable to the original 17-item version. Participants rated the extent to which each item described them, such as “If I believe in an idea, no obstacle will prevent me from making it happen” using a seven-point scale ranging from 1 (strongly disagree) to 7 (strongly agree). CFA showed that a one-factor model fitted our data well (CFI = 0.947, RMSEA = 0.071, SRMR = 0.047), Scale score was computed by averaging the 10 items and the Cronbach alpha for this scale in our sample was 0.830.

### Other variables

*Controls*: As past studies had found that gender and age affect career success (Frear et al., 2019; Jung and Takeuchi, 2016; Van der Heijden et al., 2009), we included these variables as controls in our model.

## Results

### Descriptive results

Table 1 shows the descriptive statistics, reliability coefficients and zero-order correlations among all the study variables. Zero-order correlations showed that the focal measures were all significantly associated (*p* < 0.05) to each other among all the three measurement points, ranging from 0.15 to 0.67 (absolute magnitude).

**Table 1:** Descriptive statistics, correlations, and reliabilities of measures for longitudinal data.

	Mean	SD	Age at T1	Gender	T1 CC	T1 PE	T1 CD	T2 PE	T2CD	T2 N-S fit	T2 D-A fit	T2 SCS	T2 PP	T3 PE	T3 CD	T3 N-S fit	T3 D-A fit	T3 SCS
Age at T1	24.16	1.35	–															
Gender (coded 0 = Female, 1 = Male)	0.38	0.49	0.72***	–														
T1 Career Competencies (CC)	3.63	0.49	0.13**	0.21***	(0.91)													
T1 Perceived Employability (PE)	3.30	0.67	0.08	0.18***	0.66***	(0.80)												
T1 Career Distress (CD)	2.70	0.77	−0.06	−0.12*	−0.43***	−0.48***	(0.82)											
T2 Perceived Employability (PE)	3.44	0.62	0.07	0.12*	0.57***	0.65***	−0.39***	(0.82)										
T2 Career Distress (CD)	2.71	0.78	−0.09	−0.10	−0.44***	−0.39***	0.56***	−0.50***	(0.85)									
T2 Needs-Supply Fit (N-S fit)	4.69	1.17	- 0.00	−0.04	0.36***	0.27***	−0.18***	0.41***	−0.35***	(0.83)								
T2 Demands-Abilities Fit (D-A fit)	5.04	0.97	0.00	0.03	0.41***	0.40***	−0.26***	0.53***	−0.39***	0.59***	(0.81)							
T2 Subjective Career Success (SCS)	3.45	0.68	0.06	0.08	0.45***	0.42***	−0.30***	0.56***	−0.43***	0.58***	0.51***	(0.84)						
T2 Proactive Personality (PP)	4.82	0.74	0.08	0.18**	0.47***	0.44***	−0.28***	0.55***	−0.34***	0.33***	0.43***	0.40***	(0.83)					
T3 Perceived Employability (PE)	3.40	0.61	0.03	0.10	0.47***	0.58***	−0.34***	0.65***	−0.39***	0.28***	0.40***	0.37***	0.43***	(0.80)				
T3 Career Distress (CD)	2.73	0.74	−0.07	−0.06	0.35***	0.38***	0.61***	−0.40***	0.68***	−0.30***	−0.33***	−0.34***	−0.32***	−0.38***	(0.82)			
T3 Needs-Supply Fit (N-S fit)	4.66	1.28	−0.05	−0.05	0.26***	0.27***	−0.15**	0.31***	−0.29***	0.46***	0.47***	0.40***	0.28***	0.38***	−0.35***	(0.88)		
T3 Demands-Abilities Fit (D-A fit)	5.15	1.04	−0.06	−0.04	0.31***	0.34***	−0.25***	0.40***	−0.31***	0.48***	0.66***	0.42***	0.33***	0.42***	−0.39***’	0.67***	(0.86)	
T3 Subjective Career Success (SCS)	3.51	0.73	−0.06	−0.04	0.30***	0.31***	−0.24***	0.35***	−0.34***	0.38***	0.44***	0.51***	0.31***	0.40***	−0.48***	0.62***	0.55***	(0.89)

*N* = 385 AY20 graduates who participated in survey at 3 time points - right after graduation (T1), 1 year after graduation (T2), and 2 years after graduation (T3) and were employed at either T2 or T3. ***p < 0.001, **p < 0.01; *p < 0.05 (2-tailed). Numbers in parentheses on diagonals are standardized Cronbach’s alpha.

#### Data checking

Prior to model fitting, we checked for skewness (asymmetry) and kurtosis indexes. This is an important step because we used Maximum Likelihood as the estimation method. Score distributions of all items were examined; skewness was within the acceptable range of −2 to +2, kurtosis was within the range of −4 to +4 (West et al., 1995) and histograms and normal Q–Q plots suggested no major violation of the assumption of normality. Outliers were also checked using boxplots and no outliers were found. Cronbach alpha values for all scales were above 0.70 (Nunnally and Bernstein, 1994). Although all the focal constructs were significantly correlated at the 0.001 level, multicollinearity was not a major concern as the correlations were all below 0.80 (see Judge et al., 1982), while variance inflation factor (VIF) analysis showed that all VIF values were less than five (see Hair et al., 2006). Asymmetry values ranged between −1.14 and 1.08, while kurtosis values were between −1.19 and 2.52, suggesting no violations of the normality assumption (Weston and Gore, 2006).

#### Measurement models

Table 2 indicates the fit statistics for the estimated measurement models. The hypothesized multi-factor measurement model showed more acceptable fit indices than the single factor model across all three time points. Additionally, item loadings were significant and in the expected direction on their respective latent factors. Table 3 depicts the goodness of fit statistics for the configural, and metric invariance tests. As the chi-square difference tests returned a non-significant loss of fit from configural to metric invariance (at 0.01 level of significance), it suggests metric invariance was supported for all five focal measures.

**Table 2:** Fit indices for the competing measurement models.

Time 1	Chi-sq	df	*p*	chi-sq/df	CFI	RMSEA	SRMR	AIC	BIC	sBIC
M1: one factor model	2,035.45	405	0.00	5.03	0.62	0.10	0.094	29,832.87	30,188.67	29,903.11
M2: four factors model	987.42	399.00	0.00	2.47	0.86	0.06	0.055	28,796.84	29,176.35	28,871.76

Time 2	Chi-sq	df	*p*	chi-sq/df	CFI	RMSEA	SRMR	AIC	BIC	sBIC
M1: one factor model	1,608.33	275	0.00	5.85	0.68	0.11	0.09	24,923.45	25,219.95	24,981.98
M2: five factors model	512.50	265	0.00	1.93	0.94	0.05	0.05	23,847.62	24,183.64	23,913.95

Time 3	Chi-sq	df	*p*	chi-sq/df	CFI	RMSEA	SRMR	AIC	BIC	sBIC
M1: one factor model	1,988.55	275	0.00	7.23	0.63	0.13	0.10	25,038.13	25,334.62	25,096.66
M2: five factors model	516.04	265	0.00	1.95	0.95	0.05	0.05	23,585.62	23,921.65	23,651.95

**Table 3:** Factorial invariance test.

Perceived employability	Chi-sq	df	*p*	chi-sq/df	CFI	RMSEA	SRMR	AIC	BIC	sBIC	chi-sq diff	df diff	*p*-value
Configural	408.77	132	0.00	3.10	0.90	0.07	0.07	15,401.93	15,627.26	15,446.41			
Metric	419.35	142	0.00	2.95	0.90	0.07	0.07	15,392.51	15,578.31	15,429.19	10.58	10	0.39

Career distress	Chi-sq	df	*p*	chi-sq/df	CFI	RMSEA	SRMR	AIC	BIC	sBIC	chi-sq diff	df diff	*p*-value
Configural	533.88	249	0.00	2.14	0.92	0.05	0.06	24,944.33	25,240.83	25,002.86			
Metric	552.64	263	0.00	2.10	0.92	0.05	0.06	24,935.09	25,176.24	24,982.69	18.76	14	0.17

Person-job fit	Chi-sq	df	*p*	chi-sq/df	CFI	RMSEA	SRMR	AIC	BIC	sBIC	chi-sq diff	df diff	*p*-value
Configural	85.44	48	0.00	1.78	0.98	0.05	0.04	12,677.71	12,843.74	12,710.48			
Metric	100.01	52	0.00	1.92	0.97	0.05	0.06	12,687.50	12,837.72	12,717.15	14.57	4	0.01

Subjective career success	Chi-sq	df	*p*	chi-sq/df	CFI	RMSEA	SRMR	AIC	BIC	sBIC	chi-sq diff	df diff	*p*-value
Configural	116.70	34	0.00	3.43	0.96	0.08	0.05	8,454.68	8,578.34	8,479.97			
Metric	119.31	38	0.00	3.14	0.96	0.07	0.05	8,449.29	8,557.00	8,471.32	2.61	4	0.62

Table 3 presents fit statistics for the configural and metric invariance models. The chi-square difference test indicated no significant loss of fit when constraining factor loadings (from configural to metric model), supporting metric invariance for all five focal measures. Converging evidence came from incremental fit comparisons: changes were within recommended thresholds for metric invariance (ΔCFI ≤ 0.010, ΔRMSEA ≤ 0.015, ΔSRMR ≤ 0.030), also, AIC, BIC did not increase substantially. Taken together, these results indicate that factor loadings can be treated as equal across waves (metric invariance), permitting interpretation of prospective associations in the cross-lagged panel model.

#### Cross-lagged panel modeling

**Figure 2:**
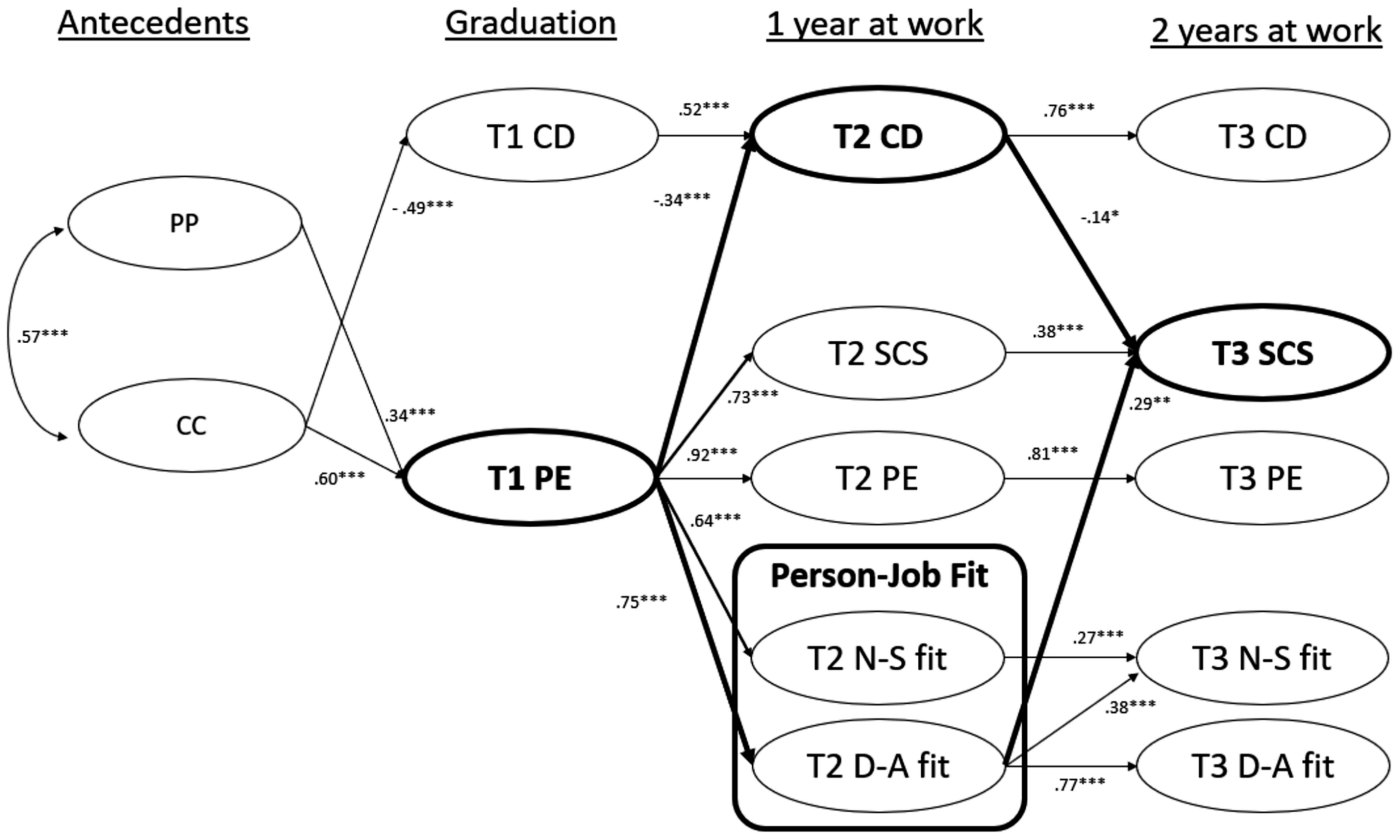
Structural model of subjective career education outcomes during university-to-work transition (*n* = 385). ***p < 0.001, **p < 0.01, *p < 0.05. Only significant standardized structural coefficients are shown. Age and gender were added to the model as controls (by regression T3SCS on them) but are not shown as their effects are not significant. PP, Proactive Personality; CC, Career Competencies; PE, Perceived Employability; CD, Career Distress; NS-fit, Need-Supplies fit; DA, Demand-Abilities fit; SCS, Subjective Career Success. Readers can refer to Table 4 for the full list of standardized parameter estimates.

Figure 2 shows the significant standardized estimates for the fitted cross-lagged model. Table 4 provides the full list of structural path estimates in the model. Model fit was acceptable based on the indices (*χ*^2^ = 5,348.935, df = 3,062, *p* < 0.001, *χ*^2^/df = 1.747, CFI = 0.859, RMSEA = 0.044, SRMR = 0.067). We observed that higher levels of pre-graduation PE predicted lower career distress (*β* = −0.34, *p* < 0.001) a year later at work, which then predicted higher SCS 2 years later at work (*β* = −0.14, *p* < 0.05). This supports Hypothesis 1. Also, pre-graduation PE predicted Need-supplies fit (*β* = 0.64, *p* < 0.001) and Demand-abilities fit (*β* = 0.75, *p* < 0.001) a year later at work. This supports Hypothesis 2. The Demand-abilities fit a year after graduation, in turn, predicted SCS the second year at work (*β* = 0.29, *p* < 0.001). The indirect effect of pre-graduation PE on SCS at T3 via Demand-abilities fit was significant, a*b = 0.22, 95% confidence intervals with 1,000 bootstrapped samples: [0.08, 0.42], which supports Hypothesis 3. Similarly, the indirect effect of pre-graduation PE on SCS at T3 via career distress was also significant, a*b = 0.05, 95% confidence interval [0.01, 0.11], which supports Hypothesis 4. In fact, Demand-abilities fit and lower career distress fully mediated the effect of pre-graduation PE on SCS (the direct effect of pre-graduation PE on SCS at T3 was not significant, *β* = 0.07, *p* = 0.71).

**Table 4:** Standardized estimates of all the direct paths in the fitted cross-lagged model.

	Standardized effect (SE)
**PP** → **PE (T1)**	0.34*** (0.07)
**CC** → **PE (T1)**	0.60*** (0.06)
PP → CD (T1)	−0.07 (0.09)
**CC** → **CD (T1)**	−0.49*** (0.09)
PE (T1) → PE (T2)	0.92*** (0.05)
PE (T1) → CD (T2)	−0.34*** (0.08)
PE (T1) → NS fit (T2)	0.64*** (0.09)
PE (T1) → DA fit (T2)	0.75*** (0.08)
PE (T1) → SCS (T2)	0.73*** (0.08)
CD (T1) → PE (T2)	0.01 (0.07)
CD (T1) → CD (T2)	0.52*** (0.09)
CD (T1) → NS fit (T2)	0.14 (0.11)
CD (T1) → DA fit (T2)	0.11 (0.10)
CD (T1) → SCS (T2)	0.04 (0.10)
**PE (T2)** → **PE (T3)**	0.81*** (0.11)
PE (T2) → CD (T3)	−0.01 (0.10)
PE (T2) → NS fit (T3)	−0.12 (0.11)
PE (T2) → DA fit (T3)	−0.08 (0.11)
PE (T2) → SCS (T3)	−0.10 (0.11)
CD (T2) → PE (T3)	−0.06 (0.07)
**CD (T2)** → **CD (T3)**	0.76*** (0.05)
CD (T2) → NS fit (T3)	−0.10 (0.07)
CD (T2) → DA fit (T3)	−0.01 (0.06)
CD (T2) → SCS (T3)	−0.14* (0.07)
NS fit (T2) → PE (T3)	−0.07 (0.07)
NS fit (T2) → CD (T3)	−0.05 (0.07)
**NS fit (T2)** → **NS fit (T3)**	0.27*** (0.08)
NS fit (T2) → DA fit (T3)	0.12 (0.07)
NS fit (T2) → SCS (T3)	0.03 (0.09)
DA fit (T2) → PE (T3)	0.08 (0.10)
DA fit (T2) → CD (T3)	−0.03 (0.09)
**DA fit (T2)** → **NS fit (T3)**	0.38*** (0.11)
**DA fit (T2)** → **DA fit (T3)**	0.77*** (0.08)
**DA fit (T2)** → **SCS (T3)**	0.29** (0.10)
SCS (T2) → PE (T3)	−0.14 (0.09)
SCS (T2) → CD (T3)	−0.01 (0.09)
SCS (T2) → NS fit (T3)	0.10 (0.10)
SCS (T2) → DA fit (T3)	0.01 (0.10)
**SCS (T2)** → **SCS (T3)**	0.38*** (0.10)
Gender → SCS (T3)	−0.02 (0.06)
Age (T1) → SCS (T3)	−0.05 (0.06)

Bolded values indicate statistically significant path coefficients (*p* < 0.05). *p < 0.05, **p < 0.01, ***p < 0.001. Standardized estimates of all structural paths are reported. PP, Proactive Personality; CC, Career Competencies; PE, Perceived Employability; CD, Career Distress; NS-fit, Need-Supplies fit; DA, Demand-Abilities fit; SCS, Subjective Career Success.

Both PE and career distress are correlated yet distinct: *r* = −0.48, −0.50 and −0.38 at T1, T2 and T3, respectively (see Table 1), suggesting that they are different work-related outcomes of university education. They are relatively stable over 2 years post-graduation (autoregressive coefficients are over 0.8 for PE, and over 0.5 for career distress in Figure 2), thus highlighting the implicit role of university education in shaping them prior to graduation. Both T1 PE and T1 career distress correlate with T2 SCS (*r* = 0.42 and *r* = −0.30, respectively, in Table 1) and T3 SCS (*r* = 0.31 and *r* = −0.24, respectively, in Table 1). These results demonstrate the predictive validity of both PE and career distress in pre-graduation as potential SCS indicators.

We included proactive personality and career competencies as antecedents in the model to represent input-based employability. Proactive personality refers to a stable disposition to take personal initiative in a broad range of activities and situations (Bateman and Crant, 1993); career competencies encompass the essential knowledge, skills, and abilities required to deliver the desired career development outcomes (Skakni et al., 2020) which can be acquired through relevant training. Proactive personality and career competencies are moderately correlated (*r* = 0.47, *p* < 0.01), thus validating trait versus acquired/learned bases for model inclusion. Both proactive personality and career competencies accounted for variance in PE (R-squared = 0.70) but only career competencies accounted for variance in career distress (R-squared = 0.29) – this validates the distinction between PE and career distress as different outcomes of university education.

## Discussion

### Theoretical contribution

This study advances vocational behavior and career development theory by conceptualizing PE as a dual psychological resource that supports both job-specific and career-wide outcomes during the university-to-work transition. Drawing on conservation of resources theory (Hobfoll, 1989, 2001; Hobfoll et al., 2018), our findings suggest that PE is associated with SCS through two distinct pathways: improved person–job fit and reduced career distress. This dual-resource framing extends conservation of resources theory by demonstrating how a single resource can be deployed across multiple domains—employment quality and psychological wellbeing—during a critical career transition. This contributes to the literature by offering a theory refinement approach (Colquitt and Zapata-Phelan, 2007) extending conservation of resources theory to the university-to-work transition context. By modeling both person–job fit and career distress as mediators of the PE–SCS relationship, we integrate traditionally siloed constructs from job and career literatures.

A dynamic lens may be particularly important during the first years after labor market entry. Although our model specifies prospective paths, the relationships may not be strictly unidirectional. For instance, reduced distress or improved person–job fit could, over time, strengthen graduates’ sense of employability, reflecting the gain cycles described in conservation of resources theory (Halbesleben et al., 2014). Empirical evidence supports this possibility. Perceived employability has been shown to shift across the graduation transition (Grosemans et al., 2023) and, in some cases, to precede later increases in career engagement (Grosemans et al., 2024). At the same time, not all antecedents are equally fluid. Broad personality traits tend to show considerable rank-order stability in young adulthood (Bleidorn et al., 2022), whereas career competencies appear more responsive to early career experiences (Grosemans and De Cuyper, 2021). For this reason, we interpret the present findings as primarily capturing between-person differences across time. Designs that explicitly separate stable individual differences from within-person change would allow a more direct test of reciprocal dynamics.

The diversified investment metaphor introduces a heuristic for strategic resource allocation across short- and long-term career goals. This framing clarifies the mechanisms through which PE operates and advances theoretical understanding of employability development during early career transitions. While our data do not directly test investment decision-making, we interpret PE as a diversified psychological investment—akin to a portfolio that balances short-term employment fit with long-term career resilience. This metaphor complements conservation of resources theory’s emphasis on resource accumulation, protection, and deployment, and aligns with career capitalist perspectives (Inkson and Arthur, 2001; Mallon, 1999). This framing offers a heuristic for understanding how graduates allocate psychological resources during the university-to-work transition: a view that PE enables individuals to invest simultaneously in securing well-fitting employment and building career resilience. Future research could examine this investment logic more directly to assess its theoretical utility and empirical validity.

Our model also responds to calls for greater integration between job and career literatures (Hall and Las Heras, 2010; Arthur et al., 1989). By modeling both person–job fit (Edwards, 1991; Cable and DeRue, 2002) and career distress (Creed et al., 2016) as mediators, we clarify how PE operates across traditionally siloed domains. This helps resolve conceptual ambiguities in employability research, where outcomes are often conflated or narrowly defined (Fugate et al., 2021).

The inclusion of proactive personality (Bateman and Crant, 1993; Seibert et al., 2001) and career competencies (Akkermans et al., 2013) as antecedents adds explanatory depth. It highlights how both trait-based and developmental resources contribute to employability, with prior research showing that career competencies enhance employability and career success through increased self-awareness and proactive career behaviors (De Vos et al., 2011). Career competencies have also been shown to support employability and buffer against career shocks, making them critical for sustainable career development among young professionals (Blokker et al., 2019). They also relate to career success through job crafting—proactive efforts to shape one’s work environment to better fit personal strengths and career goals (Akkermans and Tims, 2017). Career competencies are not static attributes but evolve during the transition from higher education to the labor market, making them especially relevant for modeling employability development in early career stages (Grosemans and De Cuyper, 2021).

### Practical implications

*For universities and career education policy*: Universities should adopt a holistic approach to career education that fosters both employability and employment outcomes. Our findings suggest that PE and career distress are stable, predictive constructs that reflect the psychological impact of university career support. Institutions should consider tracking these indicators alongside traditional metrics like job placement and salary (Jackson and Bridgstock, 2018; Tomlinson, 2012). Career services should integrate employability development (e.g., career competencies, adaptability training) with job search support. Recent research also shows that career competencies influence SCS indirectly via employability activities, and that academic satisfaction can moderate this relationship (Lo Presti et al., 2022). This supports our inclusion of career competencies as a learned resource that graduates can mobilize during the school-to-work transition. This dual-track approach aligns with the dual-resource model and prepares students for both immediate employment and long-term career sustainability (Savickas, 2013; Akkermans et al., 2021).

*For students and graduates*: Graduates often face a dilemma during the university-to-work transition: whether to focus on securing a job or investing in long-term employability. Our model suggests that both are important and interconnected. Students should be encouraged to view employability development as a strategic investment—even after securing employment. Our diversified investment metaphor offers a promising heuristic for understanding how individuals allocate psychological resources during career transitions. Much like financial investors balance risk and return across asset classes, graduates may strategically distribute time, effort, and attention across short-term employment goals and long-term career development. This metaphor therefore provides a useful lens for understanding how to balance short-term and long-term career goals; to help individuals think of employability as a strategic resource that they should invest in to navigate career uncertainty (Lo Presti and Pluviano, 2016).

*For career counselors and educators*: Career construction theory (Savickas, 2005) emphasizes adaptability and narrative identity, which complements our dual-resource model by highlighting how individuals author their career paths. Career counselors can use the dual-resource model to explain how PE contributes to both employment quality and career wellbeing. The model provides a framework for discussing how career competencies and proactive traits influence employability and distress, and how these in turn shape career outcomes. It also helps clarify the distinction between employment and employability, which is often misunderstood (Fugate et al., 2004; Römgens et al., 2020).

*For employers and HR professionals*: Recent research also highlights the role of job crafting in enhancing person–job fit and employability (Sameer and Priyadarshi, 2023). Employers can support early career success by enhancing internal mobility, offering developmental opportunities, and recognizing the role of PE in shaping employee engagement and retention. Job demands and resources have been shown to predict outcomes such as absenteeism and disengagement (Bakker et al., 2003), reinforcing the importance of fostering person–job fit and employability in organizational settings. Strengthening employees’ PE can lead to better person–job fit and lower career distress, benefiting both the individual and the organization (Cappelli and Keller, 2014; Hogan et al., 2013). Organizations should consider policies that allow employees to proactively shape their roles (e.g., job crafting), access career development resources, and engage in internal career mobility. These practices can help retain talent and foster sustainable career growth.

### Limitations and future directions

The diversified investment metaphor is intended as a heuristic and was not directly tested in the present study. Future research could adopt designs with additional waves to examine whether these constructs change within individuals over time and whether effects operate in both directions. Approaches such as random-intercept cross-lagged panel models (RI-CLPMs) or related models would be particularly useful for distinguishing stable individual differences from evolving intraindividual processes. While our study infers this logic from downstream outcomes, future research could directly examine resource allocation decisions using diary methods, experimental designs, or longitudinal tracking of career behaviors. For example, researchers could explore how individuals prioritize job search intensity versus skill development, or how PE influences trade-offs between immediate job acceptance and long-term fit. Spurk et al. (2019) emphasize the importance of studying career success as a dynamic process shaped by individual agency and contextual factors—an approach well-suited to testing the investment logic embedded in employability development. By operationalizing resource allocation as a measurable construct, future studies could validate and extend the metaphor, offering richer insights into how individuals navigate the university-to-work transition and beyond.

This model offers a theoretically grounded and empirically testable framework for understanding how employability functions as a dual resource during the university-to-work transition. It also provides a heuristic for interpreting employability development as a diversified investment in both job and career outcomes. While person–job fit and career distress differ in conceptual nature—one reflecting employment quality and the other psychological strain—they were selected as mediators to represent distinct yet complementary pathways through which PE influences career success. Despite their asymmetry, these constructs offer a balanced view of both external and internal career experiences during the university-to-work transition. Person–job fit captures the alignment between individual capabilities and job demands, whereas career distress reflects emotional challenges in career decision-making. Future research could explore more symmetrical mediators to strengthen the dual-pathway logic, such as person–career fit (Jansen and Kristof-Brown, 2006) to parallel person–job fit, or career adaptability (Rudolph et al., 2017; Zacher, 2014, 2015) as a positive counterpart to career distress. These alternatives may offer a more conceptually aligned framework for modeling employability’s impact on career outcomes.

Proactive personality and career competencies were modeled as antecedents, but their conceptual overlap with PE raises questions about temporal sequencing. Future research could explore reciprocal relationships among these constructs and examine how career education interventions shape employability development (Akkermans et al., 2024).

In studying moderators, it is useful to note that PE and career distress are shaped not only by individual resources but also by contextual factors such as social capital and core self-evaluations (Creed and Gagliardi, 2015). Those with stronger networks and positive self-perceptions are less likely to experience distress and more likely to maintain a sense of employability, even in suboptimal job conditions. Future research could explore potential moderators of the PE–SCS relationship, such as personality traits or contextual factors. Latent moderated structural equation modeling (Cheung et al., 2021) offers a promising approach for testing such interactions while accounting for measurement error.

Our sample was drawn from a single university in Singapore, a context characterized by low unemployment and high educational attainment. Singapore’s unique cultural and economic context—characterized by low unemployment and high educational attainment—may shape PE and SCS differently than in other regions. This may limit the generalizability of our findings to other cultural or labor market contexts. Additionally, we did not account for job changes, promotions, or major–job congruence, which may influence both fit and career satisfaction during the university-to-work transition (Ng and Feldman, 2014).

Our study focused on the first 2 years post-graduation. While this is a critical period, longer-term studies are needed to understand how PE and career distress evolve over time. Future research could examine how career shocks (e.g., layoffs, industry changes) affect employability perceptions and career outcomes (Blokker et al., 2019). Studies targeting mid-career professionals, retrenched workers, or those returning to education could provide valuable insights into employability dynamics across the lifespan (De Lange et al., 2021). Finally, while our model captures key psychological and employment-related constructs during the university-to-work transition, it does not account for important career events such as job changes, promotions, which may significantly influence P-J fit and SCS, particularly in dynamic labor markets. Future research should incorporate these career events to provide a more nuanced understanding of employability development and its impact on early career trajectories.

## Conclusion

This study demonstrates that PE may function as a dual psychological resource during the university-to-work transition, influencing SCS through both employment quality and psychological wellbeing. By modeling person–job fit and career distress as mediators, we clarify the mechanisms through which PE affects early career outcomes. While we do not directly test investment decisions, the metaphor of diversified investment provides a useful lens for interpreting how individuals manage competing demands and allocate psychological resources. This framing enriches conservation of resources theory and offers a bridge between vocational psychology and career development literatures. Practically, our findings suggest that universities should adopt a holistic approach to career education—one that fosters employability, reduces career distress, and prepares students for both employment and lifelong career development. Employers can support early career success by enhancing internal mobility, offering developmental opportunities, and recognizing the value of employability as a resource.

## Data Availability

The datasets presented in this article are not publicly available due to ethical and privacy considerations. Anonymized data may be made available by the corresponding author upon reasonable request, subject to conditions consistent with participant consent and institutional ethics requirements.

## Footnotes

1 The random-intercept cross-lagged panel models (RI-CLPMs) have been proposed to separate stable between-person differences from within-person dynamics (e.g., Hamaker et al., 2015). However, RI-CLPMs require three or more waves per construct; in our panel, person–job (P–J) fit and subjective career success (SCS) were measured only at T2–T3, so an RI-CLPM for the full network is not identified. We therefore used the traditional CLPM to test prospective between-person associations instead.
